# Incommensurate structures of the [CH_3_NH_3_][Co(COOH)_3_] compound

**DOI:** 10.1107/S2052252518015026

**Published:** 2019-01-01

**Authors:** Laura Canadillas-Delgado, Lidia Mazzuca, Oscar Fabelo, J. Alberto Rodriguez-Velamazan, Juan Rodriguez-Carvajal

**Affiliations:** a Diffraction Group, Institut Laue Langevin, 71, avenue des Martyrs, Grenoble 38042, France; b Centro Universitario de la Defensa de Zaragoza, Crtra. Huesca s/n, Zaragoza 50090, Spain

**Keywords:** phase transitions, incommensurate structures, formate ligand, multiferroic materials, aperiodic structures, materials science, inorganic chemistry, phase transitions, MOFs

## Abstract

The present article is devoted to the characterization of the structural phase transitions of the [CH_3_NH_3_][Co(COOH)_3_] (**1**) perovskite-like metal–organic compound through variable-temperature single-crystal neutron diffraction. At room temperature, compound **1** crystallizes in the orthorhombic space group *Pnma* (phase **I**). A decrease in temperature gives rise to a first phase transition from the space group *Pnma* to an incommensurate phase (phase **II**) at approximately 128 K. At about 96 K, this incommensurate phase evolves into a second phase with a sharp change in the modulation vector (phase **III**). At lower temperatures (*ca* 78 K), the crystal structure again becomes commensurate and can be described in the monoclinic space group *P*2_1_/*n* (phase **IV**). Although phases **I** and **IV** have been reported previously [Boča *et al.* (2004). *Acta Cryst.* C**60**, m631–m633; Gómez-Aguirre *et al.* (2016). *J. Am. Chem. Soc.* **138**, 1122–1125; Mazzuca *et al.* (2018). *Chem. Eur. J.* **24**, 388–399], phases **III** and **IV** corresponding to the *Pnma*(00γ)0*s*0 space group have not yet been described. These phase transitions involve not only the occurrence of small distortions in the three-dimensional anionic [Co(HCOO)_3_]^−^ framework, but also the reorganization of the [CH_3_NH_3_]^+^ counter-ions in the cavities of the structure, which gives rise to an alteration of the hydrogen-bonded network, modifying the electrical properties of compound **1**.

## Abstract 2

The temperature evolution of [CH_3_NH_3_][Co(COOH)_3_] on a single crystal and the crystal structure determination of intermediate incommensurate phases of [CH_3_NH_3_][Co(COOH)_3_] by means of neutron diffraction are presented.

## 1. Introduction   

The development and characterization of new materials are key challenges in condensed matter chemistry and physics. The ability of metal–organic frameworks (MOFs) to combine within the same framework different physical properties has attracted much interest in recent years (Zhu & Xu, 2014 ▸; Cui *et al.*, 2016 ▸; Lin *et al.*, 2014 ▸; Coronado & Espallargas, 2013 ▸; Liu *et al.*, 2014 ▸, 2016 ▸; Li *et al.*, 2016 ▸). These compounds constitute a promising approach for combining paradielectric, ferroelectric or antiferroelectric behaviours with long-range magnetic order. This new generation of materials are the multiferroic metal–organic frameworks (Jain *et al.*, 2009 ▸; Rogez *et al.*, 2010 ▸; Tian *et al.*, 2014 ▸). To date, most of the known multiferroic materials are purely inorganic perovskites. The most abundant representative compounds exhibiting such behaviour are, certainly, perovskite oxides with the *AB*O_3_ formula (Shang *et al.*, 2013 ▸; Van Aken *et al.*, 2004 ▸; Vrejoiu *et al.*, 2008 ▸; Rout *et al.*, 2009 ▸; Khomchenko *et al.*, 2011 ▸; Catalan & Scott, 2009 ▸). One example is the BiFeO_3_ compound, which represents a rare case with magnetic and ferroelectric ordering coexisting at room temperature (Lebeugle *et al.*, 2008 ▸). Another important material that marked the beginning of the success of this family of compounds is YMnO_3_, characterized by a multiferroic behaviour where the ferroelectric order derives from geometrical effects (Van Aken *et al.*, 2004 ▸). A more recent system in which a different magnetoelectric coupling can be found is TbMnO_3_, whereby ferroelectricity occurs as a consequence of a special kind of magnetic order (Kenzelmann *et al.*, 2005 ▸). All the multiferroic materials cited above are only inorganic-based perovskites, but a first exception was introduced in the literature by Jain *et al.* (2008 ▸) with a hybrid inorganic–organic framework material of the general formula *ABX*_3_, in which *A* is an organic cation, *B* is the metal centre and *X* is an organic bridging ligand. The presence of organic molecules contributes to the formation of hydrogen bonds that are often responsible for structural phase transitions, giving ferroelectric behaviour to the compound (Ramesh, 2009 ▸). In this context, formate-based metal–organic compounds have been revealed to exhibit a combination of both dielectric and magnetic orders (Lawler *et al.*, 2015 ▸; Qin *et al.*, 2015 ▸; Mączka, Gągor *et al.*, 2017 ▸; Mączka, Janczak *et al.*, 2017 ▸). These compounds are typically synthesized by reaction of the formate ligand with a metal salt under solvothermal conditions or by slow evaporation or diffusion techniques. Typically, these compounds present a three-dimensional framework constructed from the formate ligand and metal ions, where a counter-ion is located in the cavities. The formate ligand has the ability to mediate ferro- or antiferro-magnetic interactions between the connected metal ions, depending on its coordination mode, promoting long-range magnetic order in the framework. The paradielectric, ferroelectric or antiferrolecric order is normally achieved due to order–disorder of the counter-ion within the cavities, triggered by slight differences in the hydrogen-bonded network. Then, the adequate combination of a well known framework with different ammonium-based counter-ions could be used to favour the occurrence of structural transitions and change the weak interaction network of the sample, promoting remarkable changes in its physical properties. Hence, the possible combination of long-range magnetic order with electric order makes these compounds excellent candidates for the development of multiferroic behaviour (Wang *et al.*, 2007 ▸; Xu *et al.*, 2011 ▸; Cañadillas-Delgado *et al.*, 2012 ▸; Mączka *et al.*, 2016 ▸).

The occurrence of an order–disorder transition can involve the blocking of the libration of the counter-ion or the reorganization of these molecules in the cavities. These effects produce a symmetry breaking of the system. Sometimes a doubling of the crystallographic axes is needed in order to explain the physical nature of the phase transition (Cañadillas-Delgado *et al.*, 2012 ▸). However, more complex order–disorder transitions are also possible. The competition between atomic interactions in pure inorganic *AB*O_3_ perovskites is responsible for the occurrence of unusual instabilities, which occasionally lead to incommensurate structures (Arakcheeva *et al.*, 2017 ▸; Khalyavin *et al.*, 2015 ▸; Lin *et al.*, 2015 ▸; Arévalo-López *et al.*, 2015 ▸; Du *et al.*, 2014 ▸; Szczecinski *et al.*, 2014 ▸; Magdysyuk *et al.*, 2013 ▸). The atomic interactions responsible for the incommensurate distortions involve a counter-ion displacement, as well as antiferrodistorsive motions, mainly the tilting of the oxygen octahedron centred in the *B*-site.

Among the low number of incommensurate structures reported in the Bilbao Crystallographic Server database (Aroyo *et al.*, 2011 ▸) (149 entries of which 24 are composites), there are no examples of organic–inorganic perovskite-like systems, although a similar scenario to the pure perovskite compounds is expected (see, for example, Fütterer *et al.*, 1995 ▸). To the best of our knowledge, the closest example is the (C_6_H_11_NH_3_)_2_[PbI_4_] compound, which presents a hybrid layered perovskite-like structure (K_2_NiF_4_-type). This compound crystallizes in the orthorhombic space group *Pbca* at room temperature (RT), and below 128 K it presents a structural phase transition to the superspace group *Pca*2_1_(α½0), disturbing its physical properties (Yangui *et al.*, 2015 ▸). Furthermore, other metal–organic layer-based examples, such as *n*-propylammonium manganese chloride, have also been studied previously (Depmeier, 1981 ▸).

The refinement of modulated structures requires the use of the superspace formalism in which every structural parameter 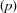, (*e.g.* atom positions, displacement parameters, occupation factors *etc*.) is described in terms of the average parameter 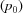 and a periodic modulation function of the internal coordinate 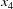. The internal coordinate is defined as 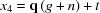, where **q** is the modulation vector, *g* is a phase reference point that, in our case, is the atomic position of the average structure, *n* defines the position of the current unit cell and *t* is the phase factor. The actual value of the parameter *p* for a particular atom in the incommensurate phase is given by 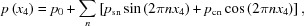
where *p*_0_ is the value of the parameter in the average structure, *p*_sn_ and *p*_cn_ are the amplitudes of the displacement modulation and *n* is the order of harmonics in the Fourier series. Then, for each structural parameter affected by modulation, it is necessary to refine *p*_0_, *p*_sn_ and *p*_cn_ in order to fully determine the incommensurate structure (Petřiček *et al.*, 2016 ▸).

Formate compounds have a predilection for the 4^12^·6^3^-cpu topology, where the formate group acts as bis-monodentate in an *anti–anti* coordination mode. This coordination gives rise to structures with medium-sized cavities, where an adequate guest molecule can be located in order to promote electric order. The occurrence of an intricate hydrogen-bonded network is mainly due to the ability of the formate anion to act as a proton acceptor. As in the case of pure inorganic compounds, the interaction between the guest molecule and the framework can be the driving force of unusual structural instabilities, promoting incommensurate structures.

The present article is devoted to the study of a series of phase transitions of the [CH_3_NH_3_][Co(HCOO)_3_] (**1**) formate compound at temperatures below the well known *Pnma* orthorhombic phase. In previous studies, a phase transition between the space group *Pnma* (at 135 K) and the monoclinic space group *P*2_1_/*n* (at 45 K) has been reported and related with changes in the electrical behaviour of compound **1** (Mazzuca *et al.*, 2018 ▸). Here, we will describe the occurrence of orthorhombic incommensurate structures upon cooling from 135 to 45 K. It should be noted that modulation parameters are usually very sensitive to defects in the basic structure model. Moreover, determination of the atomic position in each phase is fundamental to understanding the mechanism of these phase transitions. In order to consider all atoms, including the hydrogen atoms, and to establish the hydrogen-bond network, single-crystal neutron diffraction measurements at different temperatures were carried out to fully determine the modulated crystal structures.

## 2. Experimental details   

### 2.1. Sample preparation   

Aqueous solutions of CoCl_2_·6H_2_O (3 ml, 0.33 *M*), CH_3_NH_3_Cl (3 ml, 0.33 *M*) and NaHCOO (2 ml, 1.5 *M*) were mixed with 8 ml of *N*-methyl­formamide (HCONHCH_3_). The resulting solution was sealed in a Teflon-lined stainless steel vessel (43 ml), heated at 413 K for 3 d under autogenous pressure, and then cooled to room temperature. After slow cooling, pink prismatic crystals of [CH_3_NH_3_][Co(COOH)_3_] suitable for single-crystal diffraction were obtained in a yield of ∼88%. The crystals were filtered off, washed with ethanol (10 ml) and dried at room temperature. Analysis calculated for C_4_H_9_CoNO_6_ (%): C 21.24; H 4.01; N 6.20; found: C 21.37; H 4.10; N 6.22. FT–IR (cm^−1^): ν(N—H): 3118 (*sh*) and 3025 (*br*), ν(CH_3_): 2968 (*w*), 1456 (*m*) and 1418 (*m*), ν(C—H): 2875 (*m*) and 2779 (*w*), ν(NH): 2610 (*w*) and 2490 (*w*), ν(OCO): 1567 (*s*) and 1554 (*s*), ν(OCO): 1353 (*s*), 1067 (*w*), ν(C—N): 1000 (*m*) and 971 (*m*), ν(OCO): 807 (*s*).

### 2.2. Single-crystal neutron Laue diffraction measurements   

**Figure 1:**
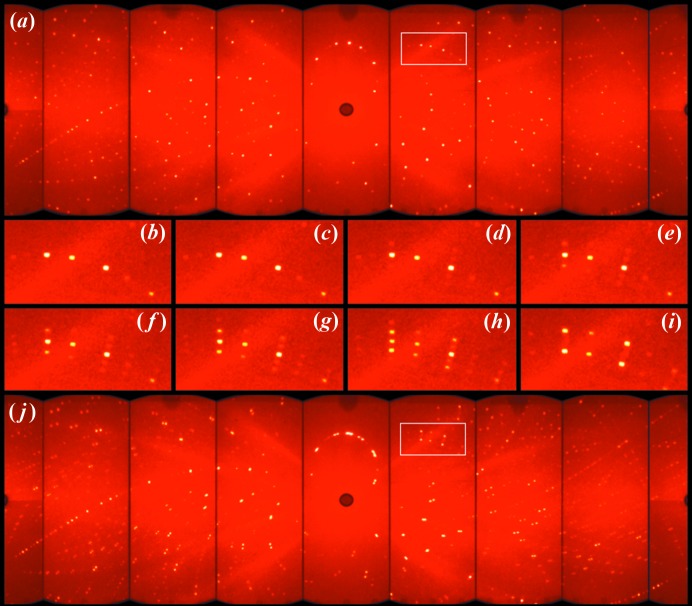
Temperature evolution of the Laue patterns: (*a*) corresponds to the orthorhombic phase collected at 138 K, (*b*)–(*i*) correspond to an enlarged area, highlighted in (*a*) and (*j*) with a white rectangle. The (*b*)–(*i*) patterns were collected at approximately 135.5, 127.5, 122.5, 114, 105, 94, 81 and 77 K, respectively. The (*j*) pattern, collected at 65 K, corresponds to the monoclinic phase. The splitting of the nuclear reflection into two twin domains is caused by the breaking of symmetry.

The Laue diffraction measurements were collected on the multiple CCD diffractomer CYCLOPS (Cylindrical CCD Laue Octagonal Photo Scintillator) at ILL (Grenoble, France) (Ouladdiaf *et al.*, 2011 ▸). The Laue pattern permits us to perform a fast exploration of the reciprocal space as a function of an external parameter. A single crystal of about 36 mm^3^ was mounted on a vanadium pin and placed in a standard orange cryostat, the diffraction patterns were recorded in the temperature range from 140 to 65 K, following a ramp of 0.1 K every 3 min. The sample was centred on the neutron beam by maximization of the intensity of several strong reflections in the *x*, *y* and *z* directions, after which, a specific orientation was selected and the temperature evolution was collected. Each Laue diffraction pattern was collected over a period of 15 min with a difference of temperature of 0.5 K. From the temperature evolution of the Laue diffraction pattern, the occurrence of several unknown phases was observed. The graphical visualization of the Laue patterns, as well as the indexing of commensurate phases, was performed using the *ESMERALDA* software developed at ILL (Fig. 1 ▸) (Rodríguez-Carvajal *et al.*, 2018 ▸).

**Figure 2:**
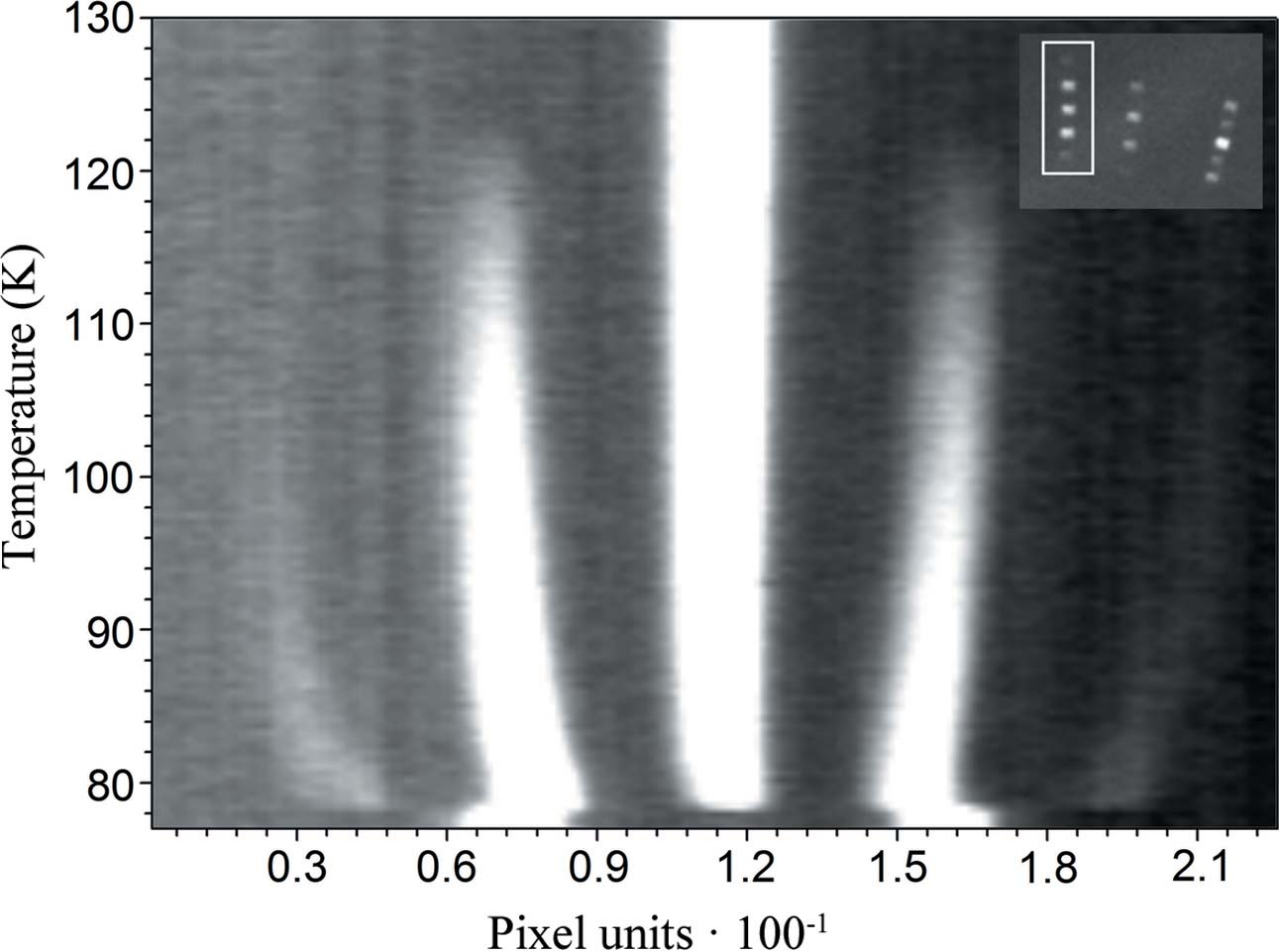
Temperature evolution of the integrated intensities of the 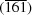 reflection in the orthorhombic phase above 128 K. Below 128 K, the first-order satellites with index 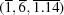 and 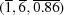, corresponding to (*hkl*) ± **q**, are observed. Below 90 K, a shift in the position of the satellites together with an increase in intensity of the second-order satellites is noticeable. This shift in the position of the reflections corresponds to the change of the wavevector from **q** = [0, 0, 0.1430 (2)] to **q** = [0, 0, 0.1247 (2)], while the change in intensity of these satellites is related to changes in the modulation amplitudes. Below 78 K, two single reflections are observed. These two reflections were indexed with the indices 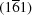 and 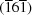, corresponding to the same diffraction plane belonging to two different twin domains. The inset highlights the region of interest used during the integration.

Although there is a notable change in the Laue diffraction pattern (Fig. 1 ▸) as a function of temperature, the preliminary specific heat measurement did not show any signal in this temperature range. Above 128 K, the Laue pattern can be indexed using the *Pnma* orthorhombic unit cell (Table 1 ▸ and Fig. 1 ▸). Between 128 and 78 K, the occurrence of satellite reflections suggests the presence of incommensurate structures. The evolution of these satellites as a function of temperature indicates a variation in the wavevector, while the change in intensity of the main and satellite reflections points to a change in the modulation amplitude and therefore a structural evolution (Fig. 2 ▸). As a result of this crystal structure evolution, below 78 K the crystal can be indexed using a commensurate monoclinic unit cell, although the presence of two twin domains is observed in the Laue diffraction pattern, in agreement with the results reported previously from single-crystal data obtained at 45 K (Mazzuca *et al.*, 2018 ▸). It should be noted that there is a clear correlation between the different phases, probably due to a group–subgroup relation.

The temperature evolution of the Laue diffraction patterns shows that the orthorhombic reflections remain almost at the same positions in the incommensurate phases; however, the intensity of these main reflections diminishes as the temperature decreases, particularly in the vicinity of the monoclinic phase transition. As shown in Fig. 2 ▸, the behaviour of the first-order satellites is the opposite; as the temperature decreases, the intensity of the satellites increases. Up to second-order satellites are observed for the strongest reflections. However, below a critical temperature, the main reflections from the orthorhombic phase and the first and higher order satellites abruptly disappear and new reflections belonging to the monoclinic phase are then observed. Although the monoclinic reflections appear close to the first-order satellites, the non-coexistence of these phases in the single-crystal measurements, together with the abrupt change from an incommensurate to a monoclinic phase, which takes place in less than 0.5 K, preclude the unambiguous definition of this last phase transition. Although different thermal treatments have been used (fast or slow cooling), at low temperature the compound always becomes a two-domain monoclinic twinned crystal. The observed twin law 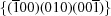 corresponds to a rotation of 180° around the crystallographic a* axis (in the monoclinic setting). It should be noted that after thermal treatment, and above 135 K, the orthorhombic commensurate phase (*Pnma*) is recovered without any evidence of damage to the sample.

### 2.3. Monochromatic single-crystal neutron diffraction measurements   

Monochromatic diffraction data were collected on the four-circle D19 diffractometer at ILL (Grenoble, France) with Cu(220)-monochromated radiation (take-off angle 2θ_M_ = 69.91°), providing neutrons with a wavelength of 1.456 Å, which is a good compromise between instrumental resolution, data completeness and the overlapping of neighbouring reflections in the incommensurate phases. The same sample used for Laue diffraction was used on D19. The sample was placed on a closed-circuit displex device, which was operated following a ramp of 2 K min^−1^. The sample and the beam stability were checked by collecting a short scan around the 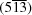 reflection. The measurement strategy consists of several ω scans with steps of 0.07° at different χ and ϕ positions. The collected data set on the incommensurate phases consists of 25 long ω scans at 122 (2), 106 (2) and 86 (2) K and 21 scans at 90 (2) K.

In previous work, two extra data sets were collected at 135 (2) and 45 (2) K in the orthorhombic and monoclinic phases, above and below the incommensurate phases. At 135 K, we collected 25 ω scans at 0.949 Å, while at 45 K, we acquired 35 ω scans at 1.454 Å (Mazzuca *et al.*, 2018 ▸). Although the crystallographic studies on these phases are not the objective of this work, a brief description of these phases will be included for completeness.

The multi-detector acquisition data software (*MAD*) from ILL was used for data collection. Unit-cell determinations were performed using *PFIND* and *DIRAX* programs, and processing of the raw data was applied using *RETREAT* and *RAFD19* programs (Duisenberg, 1992 ▸; McIntyre & Stansfield, 1988 ▸; Wilkinson *et al.*, 1988 ▸).

The calculation of possible wavevectors was carried out using the *DIRAX* program (Duisenberg, 1992 ▸) and the full data set was indexed with a single wavevector in the form **q** = γc*. Second- or third-order satellites were observed depending on the temperature range. At each temperature, the indexed wavevector was used to obtain a supercell. With this supercell, all reflections, main and satellites, were successfully integrated. The decomposition into main and satellite reflections following the superspace formalism was carried out using the new D19 software *SATELLITE*. An absorption correction was applied using *D19ABS* (Matthewman *et al.*, 1982 ▸). The structures were solved with *SUPERFLIP* (Palatinus & Chapuis, 2007 ▸) using a charge-flipping algorithm.

### 2.4. Structural determination and refinement details   

Full-matrix least-squares refinement on |*F* ^2^| using *SHELXL2014/76* as implemented in the program *WinGX* was used for structure refinement of the high-temperature phase (commensurate orthorhombic phase), while for the low-temperature data, the crystal structures were solved using the *SUPERFLIP* program (Palatinus & Chapuis, 2007 ▸). *SUPERFLIP* was used to determine the non-hydrogen-atom positions, while the hydrogen atoms were located using difference Fourier maps. The incommensurate phases were refined using the superspace formalism included in the *JANA2006* program (Petřiček *et al.*, 2016 ▸), which is currently the only available program able to handle this formalism. Exploration of the three-dimensional +1 Fourier density maps clearly indicates a displacive character close to harmonicity for both framework and counter-ion. Therefore, the displacement parameters of the different atoms were included in the refinement. After the convergence of the model, all atoms – including hydrogen atoms – were refined with anisotropic displacement parameters (ADPs). Then, the first- and second-order harmonic waves of the ADPs were introduced into the model, which take into account the changes in the crystal structure modulation. The ratios between main and satellite reflections are 0.26 for the 122 (2) and 106 (2) K data sets, and 0.11 and 0.18 for the 90 (2) and 86 (2) K data sets, respectively. The decrease of the ratio between the main and satellite reflections can be attributed to the occurrence of third-order satellite reflections in the 90 (2) and 86 (2) K data sets. There are 333 refined parameters for all incommensurate structures; a summary of the experimental and crystallographic data is given in Table 1 ▸.

The commensurate monoclinic phase collected at 45 K was refined using *JANA2006* (Petřiček *et al.*, 2016 ▸) against *F*_o_^2^ data using the full-matrix least-squares algorithm. The occurrence of a twin at this temperature was taken into account during the data refinement. The contribution of each twin domain was close to 50%. In the final refinements, all atoms, including the hydrogen atoms, were refined with ADPs. Graphical representations of all phases were produced using the program *DIAMOND* (Version 4.4.0; Brandenburg & Putz, 1999 ▸). Crystallographic data, in CIF format, for the structures of phases **II** and **III** have been deposited at the Bilbao Crystallographic Server Database with reference number 13542El8AS4.

## 3. Results and discussion   

Although the structures of compound **1** in the orthorhombic space group *Pnma* at high temperature and in the monoclinic phase (*P*2_1_/*n* space group) at low temperature have already been reported (Boča *et al.*, 2004 ▸; Gómez-Aguirre *et al.*, 2016 ▸; Mazzuca *et al.*, 2018 ▸), for the sake of clarity in the comparison of these two phases with the incommensurate phases, we will give some details about them. The description of the different phases will be given as a function of the decreasing temperature, although a similar behaviour is observed on heating.

### 3.1. Commensurate orthorhombic phase   

**Figure 3:**
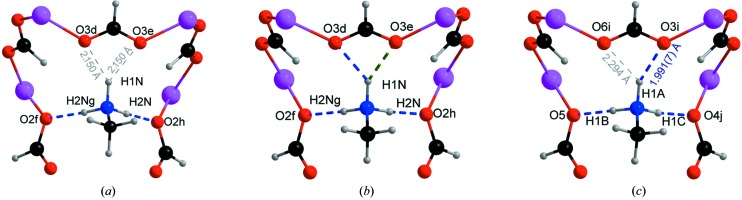
View of the possible hydrogen bonds, denoted in discontinuous blue and green lines, involving the methyl­ammonium cation in (*a*) the orthorhombic commensurate phase, (*b*) the incommensurate phases and (*c*) the commensurate monoclinic phase. The distances denoted in grey are too large to be considered as hydrogen bonds. In the incommensurate phases, the distances H1N⋯O3d and H1N⋯O3e are in the ranges 2.075–2.133, 2.009–2.132, 1.973–2.113 and 1.988–2.123 Å at 122, 106, 90 and 86 K, respectively (see details in Tables 4 and 5). Symmetry codes: 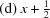, 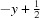, 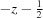; 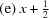, 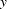, 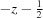; 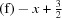, 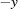, 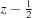; 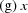, 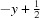, 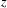; 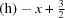, 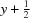, 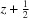; 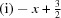, 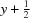, 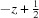; 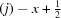, 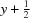, 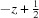.

Compound **1** crystallizes in the orthorhombic space group *Pnma* between RT and ∼128 K (phase **I**). The crystal structure consists of a three-dimensional anionic [Co(HCOO)_3_]^−^ framework with a 4^12^·6^3^-cpu perovskite-like topology (Schläfli notation), where the crystallographically independent cobalt(II) ion is located in an inversion centre and is six-coordinated in an almost ideal CoO_6_ octahedron. The cobalt(II) atoms are connected through formate ligands in an *anti*–*anti* conformation along the 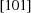, 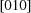 and 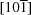 directions (*B*O_3_ sites of the Perovskite structure). In order to achieve electroneutrality, methyl­ammonium [CH_3_NH_3_]^+^ counter-ions fill the cavities of the framework (*A* sites of the Perovskite structure). This molecule sits in a mirror plane that crosses the molecule through the nitrogen and carbon atoms, parallel to the *ac* plane. At room temperature, the three hydrogen atoms connected to the carbon atom in the methyl­ammonium molecule are disordered over two different positions with occupancy factors of 0.5. However, at 135 K, before the first phase transition takes place, all three hydrogen atoms sit in single positions. There are two crystallographically independent hydrogen atoms, one is crossed by the mirror plane and the other generates the third hydrogen atom by symmetry. Note that these hydrogen atoms are not involved in any hydrogen bonding, nevertheless the hydrogen atoms connected to the nitrogen atom of the counter-ion establish two different hydrogen bonds (Fig. 3 ▸). These hydrogen bonds between the counter-ion and the three-dimensional network contribute to the stabilization of the whole structure.

### 3.2. Incommensurate structures   

The temperature dependence of the Laue diffraction patterns reveals the occurrence of two different incommensurate phases (Fig. 1 ▸). Below 128 K, the occurrence of weak new reflections close to the high-temperature orthorhombic reflections suggest a first phase transition from the orthorhombic commensurate phase (**I**), crystallized in the space group *Pnma*, to an incommensurate phase (**II**). The indexing of both main and satellite reflections gives rise to an incommensurate unit cell with the wavevector **q**, with a unique component along the *c* axis. The full data set collected at 122 (2) K suggests a wavevector of the form **q** = 0.1430 (2)c*. The same result was obtained for the full data set collected at 106 (2) K. However, for the data collected at 90 (2) and 86 (2) K, the modulation is clearly longer with wavevector **q** = 0.1247 (2)c* (Table 1 ▸). This change in modulation length, with values of [*c*/*q_z_*] of 56.98 (1), 57.02 (2), 65.46 (2) and 65.37 (2) Å for 122 (2), 106 (2), 90 (2) and 86 (2) K, respectively, suggest the presence of two different incommensurate phases. Herein, the crystal structures refined in the temperature range from 128 to 96 K will be called phase **II** and those refined between 96 and 78 K will be named phase **III**.

**Figure 4:**
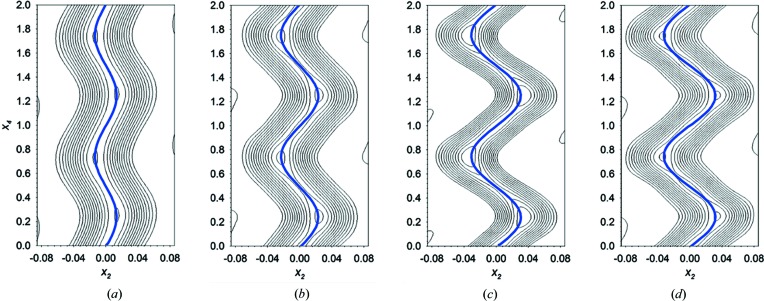
(*a*)–(*d*) Contour plot of the *x*_4_-*x*_2_ two-dimensional sections calculated fixing *x*_1_ = 0.5 and *x*_3_ = 0, corresponding to the atomic domains of the cobalt atom, with *x*_1_, *x*_2_ and *x*_3_ corresponding with *x*, *y* and *z* axes, respectively, and *x*_4_ being the parameter of modulation *t*. The cobalt contour plots obtained at 86, 90, 106 and 122 K together with the refined modulation function, denoted as a solid blue line, are represented from (*a*) to (*d*), respectively.

The refined wavevectors are close to being commensurate. The length of the *c* axis, as well as the unit-cell volume in the incommensurate phases (from 128 to 78 K), is close to seven or eight times bigger than in the commensurate orthorhombic phase (*Pnma* phase **I**). In a preliminary refinement, a supercell (assuming a strictly commensurate unit cell) was used to determine a crystal structure model. The model was solved in the three-dimensional space group *P*2_1_2_1_2_1_. However, the refined model, using the three-dimensional space group, gives rise to an unstable refinement, which can only converge after applying constraints. Therefore, even if we assume that phases **II** and **III** are almost commensurate, the quality of the data refinements is significantly better if we use the superspace group formalism. In the superspace formalism, each atom follows a curve forming the so-called ‘atomic domain’. This curve, defined as a modulation function, can be described by a periodic function characterized by a set of refined parameters. After each refinement, a four-dimensional density map can be calculated and different two-dimensional sections through a specific atom can be calculated. The shape of the modulation function must reproduce the modulation of the atomic domain in the Fourier maps, as it occurs (Fig. 4 ▸).

The determination of the superspace group was carried out with the help of the *SUPERFLIP* program for each data set from 122 (2) to 86 (2) K. Although a change in the modulation length occurs between 106 (2) and 90 (2) K, there is no breaking of symmetry and the *Pnma*(00γ)0*s*0 superspace group remains invariant in these temperature ranges (see details in Table 1 ▸).

Based on the determined superspace group, the average structure is described in the space group *Pnma*. Each independent atom in the average structure is modulated by the application of a modulation function. This modulation function should be defined for each data set; however, in the current case, all exhibit a sinusoidal behaviour, as shown in the sections of the four-dimensional Fourier maps (Fig. 4 ▸). The refined modulation functions for the cobalt atom at 86 (2), 90 (2), 106 (2) and 122 (2) K, represented as a blue curve in Fig. 4 ▸, are in good agreement with the experimental (*F*_obs_) Fourier maps. The shape of this modulation suggests a continuous character, with an increase in the displacive modulation with decreasing temperature. The refined model shows that the amplitudes of the displacive modulation have the main components along the *b* axis. A summary of the refined amplitude displacements for cobalt(II) and the carbon and nitro­gen atoms of the methyl­ammonium counter-ion, as representative of the framework and guest molecule, can be found in Tables 2 and 3. Note that, due to symmetry restrictions, the modulation of the cobalt(II) ion presents only the sine term of the Fourier coefficients (see Table 2 ▸), while for the other atoms, both sine and cosine terms are present (see Table 3 ▸).

**Figure 5:**
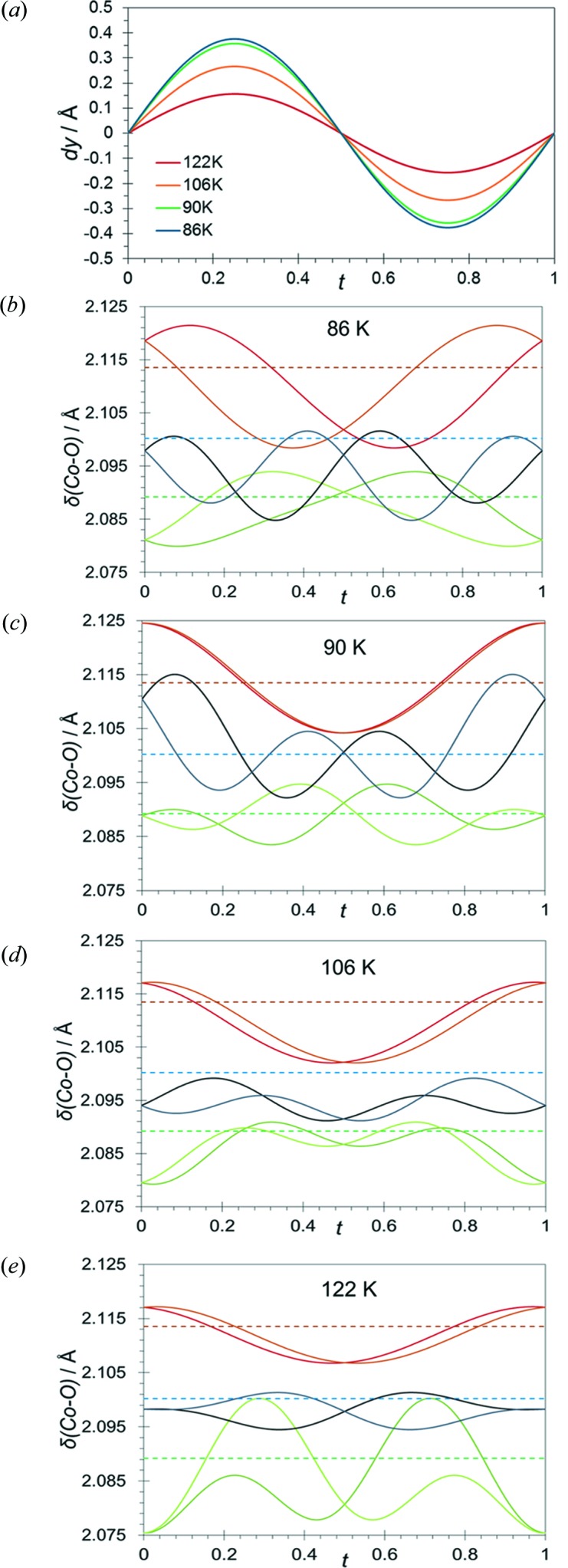
(*a*) Temperature evolution of the modulation function of the cobalt atom. (*b*)–(*e*) Modulation of the bond lengths between the cobalt and oxygen atoms at 86 and 90 K in phase **III**, and at 106 and 122 K in phase **II**. The distances Co1—O1, Co1—O1a, Co1—O2b, Co1—O2c, Co1—O3 and Co1—O3a are represented in light-green, dark-green, orange, red, black and grey continuous lines, respectively. The distances Co1—O1, Co1—O2c and Co1—O3 in the commensurate phase **I** are represented in green, brown and blue discontinuous lines, respectively. [Symmetry codes: 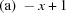, 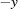, 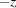; 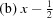, 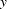, 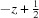; 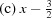, 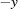, 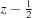.]

Based on the atomic positions present in the average structure (8*d*, 4*c* or 4*b* Wyckoff positions of the *Pnma* average structure), symmetry constraints are applied to the sine or cosine terms of the Fourier coefficients. This implies that slight tilts or distortions of the CoO_6_ octahedron are allowed by symmetry. However, these terms are notably smaller than those responsible for the modulation along the *b* axis. Therefore, the final model presents small differences between the modulation of the [Co(COOH)_3_]^−^ framework and the [CH_3_NH_3_]^+^ counter-ions. The distortion of the CoO_6_ octahedron [the maximum variation in the Co—O bond distances is *ca* 0.05 (1) Å] is much smaller than the variation of the cobalt(II) atom position due to the modulated displacement [maximum displacement = 0.375 (6) Å at 86 K] (Fig. 5 ▸).

A graphical representation of the bond-distance modulation for each Co—O bond in phases **II** and **III** is shown in Fig. 5 ▸. It is interesting to observe that at any *t* value [*t* = *x*_4_ (mod 1)], all the Co—O bond distances are in the range from 2.117 (5) to 2.075 (6) Å for phase **II** and from 2.125 (6) to 2.080 (7) Å for phase **III**, which denotes that the CoO_6_ octahedron remains an almost ideal octahedral environment in the whole range of *t*. The modulation function (Figs. 5 ▸ *b*–5*e*) shows temperature dependence, since the amplitude functions are also dependent on temperature. Furthermore, the change in the modulation wavelength from **q** = 0.1430 (2)c* to **q** = 0.1247 (2)c* produces a drastic change in the shape of the modulation function, which is in agreement with the two phases description. Finally, we would like to mention that the average displacement value, which is maximal along the *b* axis, is much larger than the variation of the bond distances due to the modulation function (Fig. 5 ▸). The same effect is observed in the [CH_3_NH_3_]^+^ counter-ions: the variation in the individual N—C, C—H and N—H bond lengths, as well as the variation in the C—N—H or H—C—N angles are much smaller than the average displacement values.

**Figure 6:**
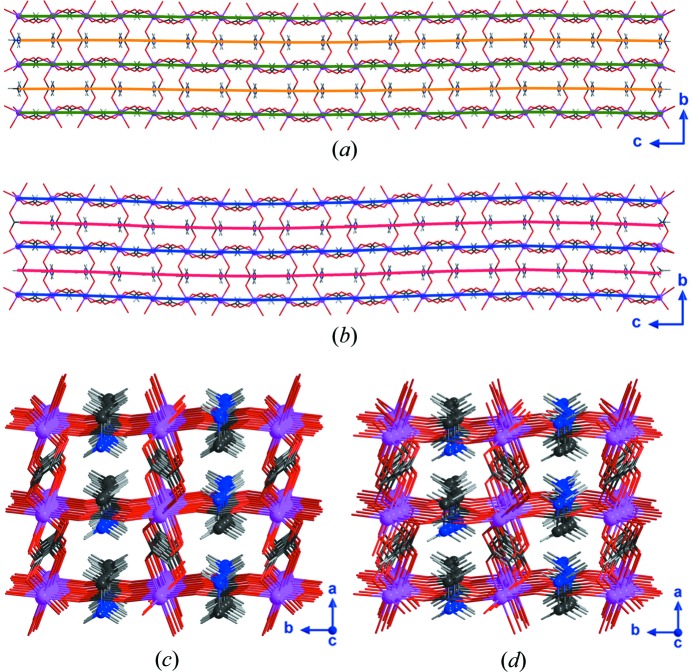
View along the *a* axis of the superstructure obtained at (*a*) 122 K and (*b*) 86 K; the solid lines are included to highlight the structural modulation, green and blue lines for the framework modulation at 122 and 86 K and orange and red for the modulation of the methylammonium cations. View along the wavevector direction of the model refined at (*c*) 122 K and (*d*) 86 K, in order to emphasize the increase of the amplitudes of the displacive modulation with decreasing temperature; all atoms have been represented as sticks except for cobalt (pink) and nitro­gen and carbon atoms (blue and black, respectively) of methylammonium. Hydrogen atoms are represented by light-grey sticks. The graphical representations were carried out considering a supercell (ten times the average unit cell along the *c* axis), in order to include at least a complete period.

A graphical description of the modulated structure for each phase, *i.e.* **II** at 122 K and **III** at 86 K, is represented in Fig. 6 ▸. The graphical representation has been carried out considering a supercell (ten times the average unit cell along the *c* axis), in order to include at least a complete period. Tables 4 ▸ and 5 ▸ show the possible hydrogen bonds defined along the incommensurate structure using a fraction of the *t* parameter for phase **II** (122 and 106 K) and **III** (90 and 86 K), respectively (Fig. 3 ▸). Along *t*, not all *D*⋯*A* distances correspond to the expected range for an ideal hydrogen bond. The distances and angles not considered to be hydrogen bonds are shown in italics. As shown, the distances elongate as a function of increasing temperature, which is compatible with an increase from thermal expansion. However, close to 106 K, we observed a slight alteration of this tendency, which is related to the proximity to the phase transition from **II** to **III**.

Slight variations in the hydrogen-bond network, which are in agreement with the subtle changes in the angles and distances, contribute significantly to the explanation of the occurrence of two incommensurate phase transitions. In Tables 4 ▸ and 5 ▸, we can see how the N1—H1n⋯O3 hydrogen bond is established via a hydrogen-bond interaction with O3d or O3e. Probably, in certain zones of the structure, the N1—H1n⋯O3d contact is established mostly, while in others it is the N1—H1n⋯O3e contact that is produced. This can be seen as a ‘flip-flop’ in the hydrogen-bond network, and therefore the competition between the intramolecular strength and the weak hydrogen-bond interactions should be responsible for the change in the modulation vectors.

### 3.3. Commensurate monoclinic phase   

Below 78 K, the satellite reflections are completely absent and the patterns can be indexed with a twinned monoclinic unit cell (phase **IV**) in the space group *P*2_1_/*n*. The ortho­rhombic (**I**) and monoclinic (**IV**) phases are related by a group–subgroup relation. The transition from phase **I** to phase **IV** involves an overall permutation of the crystallographic axes (*a* ^RT^ = −*b* ^LT^, *b* ^RT^ = −*c* ^LT^ and *c* ^RT^ = *a* ^LT^) following the next transformation matrix 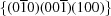, together with a change in the β angle of 1.89°. The relation between the two twin domains is a rotation of *ca* 180° about the a* axis (in the monoclinic setting). Although these two phases are separated by two orthorhombic incommensurate phases, the unit-cell volume of each phase remains almost invariable, with a slight decrease of the unit-cell volume due to the thermal contraction. It should be noted that this thermal contraction is notably anisotropic. While the *a* and *b* axes (defined in the ortho­rhombic phase) decrease continuously with temperature, with a compression of 1.5 and 0.5%, respectively, the *c* axis, which agrees with the direction of the incommensurate wavevector, increases in length by approximately 0.8%.

Although the topology of compound **1** in phase **IV** remains invariable, at low temperature there are two crystallographically independent cobalt(II) atoms (Co1 and Co2) because of the loss of symmetry operations in the transition from the orthorhombic to the monoclinic space group. Both cobalt atoms sit on inversion centres and are six-coordinated in an almost ideal CoO_6_ octahedron. Each Co1 atom is bonded to six Co2 atoms and every Co2 atom is also surrounded by six Co1 atoms, all of them connected through formate ligands in an *anti*–*anti* manner along the 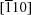, 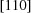 and 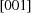 directions, building an octahedral perovskite-like framework. In the cavities of the three-dimensional structure, the methylammonium counter-ion is no longer located in a mirror plane, which means that six crystallographically independent hydrogen atoms are observed in this low-temperature phase. These variations in the structure with respect to the orthorhombic phase, together with the thermal contraction, imply changes in the hydrogen-bonded network. While in phase **I** there exists two hydrogen bonds between the guest molecule and the host framework, at low temperature the three hydrogen atoms connected to the nitrogen atom of the counter-ion establish three hydrogen bonds (Fig. 3 ▸).

## 4. Conclusions   

The crystal structure analysis of compound **1** has revealed three different phase transitions between RT and 45 K. At RT, it crystallizes in the orthorhombic space group *Pnma*. Upon cooling, at around 128 K, compound **1** undergoes a phase transition from the commensurate orthorhombic phase (**I**) to the orthorhombic incommensurate phase (**II**), crystallized in the *Pnma*(00γ)0*s*0 space group with **q** = 0.1430 (2)c*. Below 96 K, a second orthorhombic incommensurate phase was observed. The change towards the wavevector **q** = 0.1247 (2)c* involves an elongation of the modulation length. Moreover, the amplitudes of the displacive modulation increase with decreasing temperature, having the main components along the *b* axis. The evolution from the commensurate high-temperature phase to the incommensurate phases is ‘continuous’, which is compatible with a ‘displacive’ phase transition. Moreover, the modulation of the incommensurate waves is not frozen within each incommensurate phase, as the intensity of the satellites changes with temperature. This suggests a complex scenario with a ‘sluggish’ or ‘partly first-order’ transition, which can also explain the lack of signal in specific heat measurements. Furthermore, the shape of the previously reported relative permittivity curve, which shows a continuous decrease, suggests that the structural phase transition occurs in a broad temperature range. The unexpected shape of this curve prompted us to study the temperature evolution of this compound. The order–disorder phase transition, due to the hydrogen-bond reorientation, shows a jump in the permittivity curves similar to those observed for the [NH_4_][Zn(HCOO)_3_] compound (Xu *et al.*, 2010 ▸) or [NH_2_(CH_3_)_2_]_n_[Fe^III^Fe^II^(HCOO)_6_]_*n*_ (Cañadillas-Delgado *et al.*, 2012 ▸). However, this curve reminds us of the occurrence of a slight reorientation of the methyl­ammonium ions into the cavities or a small distortion of the framework. After the temperature evolution studies, this scenario has been confirmed due to the existence of incommensurate structures. The occurrence of modulated structures produces a reorganization of the existing electric dipoles, due to the continuous variation in the amplitude of the displacive modulation. Therefore, the shape in the relative permittivity curve is compatible with the slight variations in the electric dipoles due to the temperature evolution of the crystal structure.

This compound presents an intricate hydrogen-bonded network, mainly due to the ability of the formate anion to act as a proton acceptor. This fact, together with the presence of the methylammonium counter-ion in the cavities, which acts as an excellent proton donor, gives rise to a system where the competition between weak interactions produces crystal structures very close in energy. Small changes in the hydrogen-bonded network can trigger structural phase transitions, giving rise to incommensurate phases.

Below 78 K, the orthorhombic incommensurate phase becomes a monoclinic phase, resulting in a twinned crystal. The two twin domains are related by a rotation of 180° along the a* axis (in the monoclinic setting). This axis corresponds with the *c* axis in the orthorhombic phase, hence the one that becomes incommensurate between 128 and 78 K. As observed in the Laue patterns, the phase transition between the incommensurate phase [*Pnma*(00γ)0*s*0 space group with **q** = 0.1247 (2)c*] and the monoclinic phase is abrupt and therefore compatible with a first-order character. The indexing of the pattern in both phases suggests that the separation into two domains is caused by the enhancement of the amplitude of the displacement modulation in the incommensurate phase. When the amplitudes of modulation are large enough, the lower-energy structure is no longer the incommensurate structure, and a breaking of symmetry is needed to decrease the energy, giving rise to a monoclinic structure. The breaking of symmetry gives rise to a twinned crystal, with both twin domains related by the lost symmetry elements that are no longer present in the monoclinic space group *P*2_1_/*n*.

The hydrogen-bonded network between the methylammonium and the carboxylate oxygen atoms in the incommensurate structure shows a different behaviour than in orthorhombic phase **I**. Two of the three hydrogen atoms of the NH_3_ group establish hydrogen bonds, while the third fluctuates between two oxygen atoms from the same formate ligand (Fig. 3 ▸), giving rise to a ‘flip-flop’ behaviour. The modulation in the hydrogen-bond interactions suggests that competition between these interactions is responsible for the change in the modulation vector. The analysis of the hydrogen-bond interactions in the monoclinic phase shows that the three hydrogen atoms of the NH_3_ group are involved in hydrogen bonding, therefore the methylammonium counter-ions are better anchored into the framework cavities, giving rise to a more stable structure.

## Supplementary Material

Crystal structure: contains datablock(s) global, I, II, III, IV. DOI: 10.1107/S2052252518015026/lt5013sup1.cif

Temperature evolution of the [CH3NH3][Co(HCOO)3] metal-organic compound. URL: https://doi.org/10.5291/ILL-DATA.5-15-617

Structure factors: contains datablock(s) global, I. DOI: 10.1107/S2052252518015026/lt5013Isup2.hkl

Structure factors: contains datablock(s) global, I. DOI: 10.1107/S2052252518015026/lt5013IIsup3.hkl

Structure factors: contains datablock(s) global, I. DOI: 10.1107/S2052252518015026/lt5013IIIsup4.hkl

Structure factors: contains datablock(s) global, I. DOI: 10.1107/S2052252518015026/lt5013IVsup5.hkl

13542El8AS4

CCDC references: 1874920, 1874921, 1874922, 1874923

## Figures and Tables

**Table 1:** Experimental and crystallographic data of compound **1**, measured on the single-crystal neutron diffractometer D19 and refined with *JANA2006*

Chemical formula	C_4_H_9_CoNO_6_	C_4_H_9_CoNO_6_	C_4_H_9_CoNO_6_	C_4_H_9_CoNO_6_
*M*	226.05	226.05	226.05	226.05
Superspace group	*Pnma*(00γ)0*s*0	*Pnma*(00γ)0*s*0	*Pnma*(00γ)0*s*0	*Pnma*(00γ)0*s*0
*T* (K)	122 (2)	106 (2)	90 (2)	86 (2)
*a* (Å)	8.2674 (2)	8.2556 (2)	8.2702 (3)	8.2548 (3)
*b* (Å)	11.6600 (4)	11.6519 (3)	11.6766 (4)	11.6547 (6)
*c* (Å)	8.1483 (2)	8.1508 (3)	8.1631 (6)	8.1521 (3)
*V* (Å^3^)	785.48 (4)	784.05 (4)	788.29 (7)	784.29 (6)
*Z*	4	4	4	4
Modulation vector (**q**)	0.1430 (2)c*	0.1430 (2)c*	0.1247 (2)c*	0.1247 (2)c*
ρ_calc_ (mg m^−3^)	1.9115	1.915	1.9047	1.9144
λ (Å)	1.4569	1.4569	1.4569	1.4569
μ, (mm^−1^)	0.2417	0.2417	0.2417	0.2417
*R*_1_, *I* > 3σ(*I*) (all)	0.0880 (0.1189)	0.0836 (0.1012)	0.1030 (0.1449)	0.1099 (0.1469)
*wR*_2_, *I* > 3σ(*I*) (all)	0.1048 (0.1067)	0.0965 (0.0982)	0.1293 (0.1326)	0.1297 (0.1326)
Main reflections: *R*_1_, *I* > 3σ(*I*) (all)	0.0832(0.0835)	0.0828(0.0833)	0.0880(0.0909)	0.0897(0.0943)
Main reflections: *w*R**_2_, *I* > 3σ(*I*) (all)	0.0984(0.0985)	0.0964(0.0965)	0.0969(0.0971)	0.1124(0.1128)
First-order satellites: *R*_1_, *I* > 3σ(*I*) (all)	0.0970(0.1231)	0.0761(0.0847)	0.0990(0.1094)	0.1027(0.1142)
First-order satellites: *w*R**_2_, *I* > 3σ(*I*) (all)	0.1155(0.1176)	0.0924(0.0936)	0.1442(0.1454)	0.1291(0.1301)
Second-order satellites: *R*_1_, *I* > 3σ(*I*) (all)	0.1553(0.6551)	0.1272(0.2727)	0.1297(0.2096)	0.1650(0.2369)
Second-order satellites: *w*R**_2_, *I* > 3σ(*I*) (all)	0.1660(0.2329)	0.1225(0.1368)	0.1276(0.1343)	0.1568(0.1623)
Third-order satellites: *R*_1_, *I* > 3σ(*I*) (all)	–	–	0.2758(0.6862)	0.3837(0.7697)
Third-order satellites: *w*R**_2_, *I* > 3σ(*I*) (all)	–	–	0.2936(0.3658)	0.3609(0.4140)
Absorption correction	Numerical	Numerical	Numerical	Numerical
Independent reflections	3543	3544	4617	4920
No. of main reflections	734	735	473	745
No. of first-order satellite reflections	1330	1330	1336	1340
No. of second-order satellite reflections	1479	1479	1470	1491
No. of third-order satellite reflections	–	–	1338	1344

**Table 2:** Amplitude displacements of the cobalt atom (sine term)

	122 (K)	106 (K)	90 (K)	86 (K)
*x*	−0.0011 (5)	−0.0022 (5)	−0.0027 (7)	−0.0034 (7)
*y*	0.0134 (4)	0.0229 (4)	0.0306 (6)	0.0322 (5)
*z*	−0.0004 (5)	0.0004 (5)	0.0016 (8)	0.0000 (6)

**Table 3:** Amplitude displacement for the sine and cosine terms of the first order of the harmonics in the Fourier series corresponding to the N and C atoms from the dimethylammonium cation

		122 K	106 K	90 K	86 K
N1 cos	*x*	0	0	0	0
	*y*	0.00339 (13)	0.00602 (13)	0.00763 (16)	0.00747 (17)
	*z*	0	0	0	0
N1 sin	*x*	0	0	0	0
	*y*	0.01261 (13)	0.02153 (13)	0.02946 (18)	0.03089 (18)
	*z*	0	0	0	0
C3 cos	*x*	0	0	0	0
	*y*	0.00067 (19)	0.00151 (18)	0.0019 (2)	0.0015 (3)
	*z*	0	0	0	0
C3 sin	*x*	0	0	0	0
	*y*	0.01261 (19)	0.02164 (19)	0.0297 (3)	0.0311 (3)
	*z*	0	0	0	0

**Table 4:** Selected distances (Å) and angles (°) involving the ammonium group of compound **1** at 122 and 106 K (phase **II**) Distances and angles further from the ideal hydrogen-bond geometry are emphasized in italics.

	N1—H1n⋯O3d			N1—H1n⋯O3e			N1—H2n⋯O2f		
*t*	H⋯*A*	*D*⋯*A*	*D*—H⋯*A*	H⋯*A*	*D*⋯*A*	*D*—H⋯*A*	H⋯*A*	*D*⋯*A*	*D*—H⋯*A*
122 K									
0.0	2.080 (11)	2.992 (6)	153.1 (6)	*2.247 (11)*	*3.073 (5)*	*140.6 (5)*	1.835 (9)	2.860 (3)	173.4 (9)
0.1	2.075 (11)	3.007 (6)	150.1 (7)	*2.224 (11)*	*3.077 (6)*	*139.8 (5)*	1.837 (9)	2.858 (3)	174.0 (9)
0.2	2.106 (11)	3.030 (6)	144.9 (7)	*2.178 (11)*	*3.064 (6)*	*140.3 (6)*	1.835 (9)	2.860 (3)	174.9 (9)
0.3	*2.166 (11)*	*3.048 (6)*	*142.2 (6)*	2.133 (11)	3.032 (6)	144.4 (6)	1.829 (9)	2.860 (3)	174.4 (9)
0.4	*2.225 (11)*	*3.061 (6)*	*141.5 (5)*	2.101 (11)	3.001 (6)	150.5 (6)	1.821 (9)	2.856 (3)	172.0 (9)
0.5	*2.247 (11)*	*3.073 (5)*	*140.6 (5)*	2.080 (11)	2.992 (6)	153.1 (6)	1.815 (9)	2.850 (3)	170.7 (9)
0.6	*2.224 (11)*	*3.077 (6)*	*139.8 (5)*	2.075 (11)	3.007 (6)	150.1 (7)	1.814 (9)	2.848 (3)	172.1 (9)
0.7	*2.178 (11)*	*3.064 (6)*	*140.3 (6)*	2.106 (11)	3.030 (6)	144.9 (7)	1.818 (9)	2.852 (3)	174.4 (9)
0.8	2.133 (11)	3.032 (6)	144.4 (6)	*2.166 (11)*	*3.048 (6)*	*142.2 (6)*	1.824 (9)	2.859 (3)	175.0 (8)
0.9	2.101 (11)	3.001 (6)	150.5 (6)	*2.225 (11)*	*3.061 (6)*	*141.5 (5)*	1.830 (9)	2.861 (3)	174.2 (9)
106 K									
0.0	2.009 (8)	2.967 (4)	153.9 (5)	*2.288 (7)*	*3.111 (4)*	*136.1 (4)*	1.842 (7)	2.868 (3)	174.2 (7)
0.1	2.022 (8)	2.985 (4)	152.6 (5)	*2.262 (7)*	*3.110 (4)*	*137.4 (4)*	1.839 (7)	2.865 (3)	174.2 (7)
0.2	2.097 (8)	3.018 (4)	148.2 (5)	*2.206 (7)*	*3.074 (4)*	*141.4 (5)*	1.831 (7)	2.860 (3)	173.9 (7)
0.3	*2.195 (8)*	*3.053 (4)*	*142.5 (5)*	2.132 (8)	3.021 (4)	146.7 (5)	1.826 (7)	2.855 (3)	172.9 (7)
0.4	*2.267 (7)*	*3.087 (4)*	*138.0 (4)*	2.057 (8)	2.978 (4)	151.5 (5)	1.824 (7)	2.849 (3)	171.7 (7)
0.5	*2.288 (7)*	*3.111 (4)*	*136.1 (4)*	2.009 (8)	2.967 (4)	153.9 (5)	1.821 (7)	2.842 (3)	171.6 (7)
0.6	*2.262 (7)*	*3.110 (4)*	*137.4 (4)*	2.022 (8)	2.985 (4)	152.6 (5)	1.816 (7)	2.839 (3)	172.7 (7)
0.7	*2.206 (7)*	*3.074 (4)*	*141.4 (5)*	2.097 (8)	3.018 (4)	148.2 (5)	1.815 (7)	2.845 (3)	174.0 (7)
0.8	2.132 (8)	3.021 (4)	146.7 (5)	*2.195 (8)*	*3.053 (4)*	*142.5 (5)*	1.823 (7)	2.856 (3)	174.4 (7)
0.9	2.057 (8)	2.978 (4)	151.5 (5)	*2.267 (7)*	*3.087 (4)*	*138.0 (4)*	1.835 (7)	2.866 (3)	174.4 (7)

Symmetry codes: 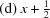, 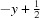, 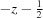; 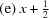, 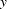, 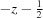; 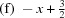, 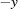, 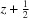.

**Table 5:** Selected distances (Å) and angles (°) involving the ammonium group of compound **1** at 90 and 86 K (phase **III**) Distances and angles further from the ideal hydrogen-bond geometry are emphasized in italics.

	N1—H1n⋯O3d			N1—H1n⋯O3e			N1—H2n⋯O2f		
*t*	H⋯*A*	*D*⋯*A*	*D*—H⋯*A*	H⋯*A*	*D*⋯*A*	*D*—H⋯*A*	H⋯*A*	*D*⋯*A*	*D*—H⋯*A*
90 K									
0.0	1.973 (12)	2.944 (6)	156.3 (7)	*2.315 (11)*	*3.118 (6)*	*134.0 (5)*	1.845 (9)	2.879 (3)	173.3 (8)
0.1	2.007 (12)	2.963 (6)	154.2 (7)	*2.303 (11)*	*3.122 (6)*	*136.0 (5)*	1.837 (9)	2.877 (3)	173.7 (8)
0.2	2.089 (12)	3.015 (6)	147.9 (6)	*2.228 (11)*	*3.099 (6)*	*140.9 (6)*	1.828 (9)	2.868 (3)	174.9 (8)
0.3	*2.186 (11)*	*3.066 (6)*	*140.8 (6)*	2.113 (12)	3.043 (6)	147.2 (6)	1.827 (9)	2.858 (3)	174.7 (8)
0.4	*2.269 (11)*	*3.099 (6)*	*135.7 (6)*	2.012 (12)	2.977 (6)	153.3 (7)	1.830 (9)	2.849 (3)	172.2 (8)
0.5	*2.315 (11)*	*3.118 (6)*	*134.0 (5)*	1.973 (12)	2.944 (6)	156.3 (7)	1.823 (9)	2.841 (3)	169.8 (8)
0.6	*2.303 (11)*	*3.122 (6)*	*136.0 (5)*	2.007 (12)	2.963 (6)	154.2 (7)	1.808 (9)	2.837 (3)	170.3 (8)
0.7	*2.228 (11)*	*3.099 (6)*	*140.9 (6)*	2.089 (12)	3.015 (6)	147.9 (6)	1.803 (9)	2.841 (3)	173.4 (8)
0.8	2.113 (12)	3.043 (6)	147.2 (6)	*2.186 (11)*	*3.066 (6)*	*140.8 (6)*	1.817 (9)	2.855 (3)	175.7 (8)
0.9	2.012 (12)	2.977 (6)	153.3 (7)	*2.269 (11)*	*3.099 (6)*	*135.7 (6)*	1.837 (9)	2.870 (3)	174.6 (8)
86 K									
0.0	1.988 (8)	2.957 (5)	155.2 (6)	*2.323 (8)*	*3.129 (5)*	*133.9 (5)*	1.848 (9)	2.875 (3)	172.4 (8)
0.1	2.010 (8)	2.974 (5)	154.1 (6)	*2.292 (8)*	*3.123 (5)*	*136.4 (5)*	1.843 (9)	2.869 (3)	173.6 (8)
0.2	2.099 (8)	3.017 (5)	148.3 (6)	*2.218 (8)*	*3.085 (5)*	*141.5 (5)*	1.834 (9)	2.859 (3)	174.8 (8)
0.3	*2.212 (8)*	*3.067 (5)*	*140.8 (5)*	2.123 (8)	3.028 (5)	147.4 (6)	1.825 (9)	2.849 (3)	174.2 (8)
0.4	*2.297 (8)*	*3.108 (5)*	*135.4 (5)*	2.035 (8)	2.977 (5)	152.3 (6)	1.817 (9)	2.840 (3)	173.0 (8)
0.5	*2.323 (8)*	*3.129 (5)*	*133.9 (5)*	1.988 (8)	2.957 (5)	155.2 (6)	1.809 (9)	2.835 (3)	171.7 (8)
0.6	*2.292 (8)*	*3.123 (5)*	*136.4 (5)*	2.010 (8)	2.974 (5)	154.1 (6)	1.807 (9)	2.834 (3)	171.5 (8)
0.7	*2.218 (8)*	*3.085 (5)*	*141.5 (5)*	2.099 (8)	3.017 (5)	148.3 (6)	1.816 (9)	2.843 (3)	172.9 (8)
0.8	2.123 (8)	3.028 (5)	147.4 (6)	*2.212 (8)*	*3.067 (5)*	*140.8 (5)*	1.831 (9)	2.858 (3)	174.4 (8)
0.9	2.035 (8)	2.977 (5)	152.3 (6)	*2.297 (8)*	*3.108 (5)*	*135.4 (5)*	1.844 (9)	2.871 (3)	173.3 (8)

Symmetry codes: 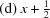, 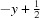 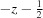; 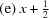, 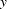, 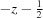; 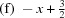, 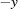, 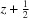.
